# Conv-ViT: A Convolution and Vision Transformer-Based Hybrid Feature Extraction Method for Retinal Disease Detection

**DOI:** 10.3390/jimaging9070140

**Published:** 2023-07-10

**Authors:** Pramit Dutta, Khaleda Akther Sathi, Md. Azad Hossain, M. Ali Akber Dewan

**Affiliations:** 1 Department of Electronics and Telecommunication Engineering, Chittagong University of Engineering & Technology, Chattogram 4349, Bangladesh; pramitduttaanik@gmail.com (P.D.); sathi.ete@cuet.ac.bd (K.A.S.); azad@cuet.ac.bd (M.A.H.); 2 School of Computing and Information Systems, Faculty of Science and Technology, Athabasca University, Athabasca, AB T9S 3A3, Canada

**Keywords:** retinal disease, classification, hybrid feature, Inception-V3, ResNet-50, vision transformer

## Abstract

The current advancement towards retinal disease detection mainly focused on distinct feature extraction using either a convolutional neural network (CNN) or a transformer-based end-to-end deep learning (DL) model. The individual end-to-end DL models are capable of only processing texture or shape-based information for performing detection tasks. However, extraction of only texture- or shape-based features does not provide the model robustness needed to classify different types of retinal diseases. Therefore, concerning these two features, this paper developed a fusion model called ‘Conv-ViT’ to detect retinal diseases from foveal cut optical coherence tomography (OCT) images. The transfer learning-based CNN models, such as Inception-V3 and ResNet-50, are utilized to process texture information by calculating the correlation of the nearby pixel. Additionally, the vision transformer model is fused to process shape-based features by determining the correlation between long-distance pixels. The hybridization of these three models results in shape-based texture feature learning during the classification of retinal diseases into its four classes, including choroidal neovascularization (CNV), diabetic macular edema (DME), DRUSEN, and NORMAL. The weighted average classification accuracy, precision, recall, and F1 score of the model are found to be approximately 94%. The results indicate that the fusion of both texture and shape features assisted the proposed Conv-ViT model to outperform the state-of-the-art retinal disease classification models.

## 1. Introduction

According to the statistics of 2019, over 2.2 billion people suffer from different eye diseases that result in serious vision impairment and partial or full blindness [1]. One of the main reasons for vision impairment is age-related macular degeneration (AMD). Several categories of AMD are found at present that individually contains unique characteristics and effect. Among these, the most well-known categories of AMD are the wet and dry classes. The dry class of AMD also happens in three stages named early, intermediate, and late [2]. From the wet class of AMD, choroidal neovascularization (CNV) causes severe vision impairment and hemorrhage. Consequently, the macula and photoreceptor-dense area are affected, which may cause blindness as this area is responsible for high-resolution vision. Every year in the USA, about 2 million people are detected with CNV [3]. On the contrary, diabetic macular edema (DME) is a type of disease that affects patients with diabetes and is related to the thickening of muscle which can be considered a complication of diabetic retinopathy. A study showed that 7.5 million people aged 40 years or older suffer from DME [4]. Another class of AMD called DRUSEN is a type of intermediate-age-related macular degeneration that affects 125 μm or more diameter of the macula, which is a region of 3000 μm centered on the foveolar in either or both eyes. From the statistical evaluation, every year, more than 7 million people in the USA are affected by the DRUSEN class [5]. If AMD can be detected earlier, complications can be avoided. For this reason, faster and more accurate detection of types of AMD plays a significant role in terms of preventing complications [2]. One of the most common approaches to detect types of AMD is doing a test called optical coherence tomography (OCT), which is a medical imaging technique where a special machine takes photos of the inside of the eye that detects back reflection from different levels of biological tissue which later forms a two or three-dimensional structural images [6]. The clinician usages OCT images to detect the types of AMD and their severity. The detection of types of AMD from these pictures is based on an algorithmic approach where handcrafted segmentation is followed by the classification of each segmented object using a statistical classifier, including a machine learning algorithm, and finally, classifying the images. This approach is not only suspected of error but also requires many skilled people. Moreover, refining and tuning a machine-learning model with handcrafted segmentation is a time-consuming and computationally expensive task [7]. For this reason, with the advancement of computer vision technology, the detection process has evolved a lot. Instead of a handcrafted feature extraction method, the automated detection process is developed to reduce error, time, and human intervention in performing the task.

In this case, several research works have been conducted to perform retinal eye disease classification based on deep learning (DL) methods. For instance, Khan et al. [8] proposed an ensemble model of ResNet50 and InceptionResNetV2 to detect ocular diseases from enhanced fundus images, and before training the model, the adaptive equalization technique was utilized to improve local contrast by modifying the intensity distribution adaptive with an object. The model achieved an accuracy of 82.05% by extracting only texture-based information from the pre-trained ensemble model. In another study, an ensemble of three models, including Inception-V3, InceptionResNetV2, and Xception, was proposed by Zhang et al. [9]. The model training was initiated by performing six preprocessing techniques, such as histogram equalization (HE), adaptive histogram equalization (AHE), intensity rescaling, gamma correction, sigmoid adjustment, and limited contrast AHE (CLAHE). After that, two types of classifiers, named deep convolutional neural network (DCNN) and deep full connection network (DFNN), were used for the final classification with an accuracy of 95.42%, which is also a result of texture-based information processing. Moreover, Wijesinghe et al. [10] proposed a transfer learning-based ensemble model consisting of DenseNet-201, ResNet-18, and VGG-16. The background removal, resolution optimization, and resizing were performed as image preprocessing to make the dataset more optimized for training. Then, techniques called global average pooling (GAP) and singular value decomposition (SVD) were used to predict a single class that combined the prediction from all the models. The extraction of only texture-based information results in an accuracy of 98.69% for the transfer learning-based ensemble model. In one of the studies, Gordon et al. [11] designed an ensemble model with the combination of two customized convolutional neural networks (CNN) to reduce variance. Moreover, the inclusion of a median filter reduces the sparkle noise. After noise removal, data augmentation is employed for training the model. The work demonstrated the trade-off between training time and classification performance and the effect of batch size on it. For model evaluation, different ensemble techniques such as majority voting, weighted averaging, and stacking were implemented with an accuracy of 99.48%, 99.47%, and 99.51%, respectively.

In contrast, Hendria et al. [12] proposed a model combining transformer and CNN models to detect objects in unmanned aerial vehicles (UAV) imagery. The Swin and DectectoRS with ResNet backbone were combined to extract the performance of the transformer as shaped-based information and CNN as texture-based information. For image preprocessing, augmentation techniques like random horizontal flip with a probability of 0.5 were used. The individual models were trained separately using identical training sets, and later, the predictions were combined to obtain the final prediction. The precision of this implemented system varied from 38.30% to 63.29%, and the recall varied from 1.42% to 56.43%. In one study, Shen et al. [13] implemented a stacked ensemble model called ‘CviT’ with a combination of convolution and transformer networks to detect movement classification. Before feeding to the CviT model, the sliding window-based preprocessing technique was utilized to divide the image into patches. For better generalization of model classification, a convolution layer was deployed, followed by the transformer model, which resulted in a classification accuracy of 83.47% and 84.09% on two different datasets. In a separate study, Aldahoul et al. [14] proposed an ensemble model with a combination of transformers to encode the retinal image. Several augmentation techniques, such as random flipping of horizontal and vertical and 360° rotation, were performed to improve the training process. The images were also rescaled between (0, 1) and cropped to remove the black border. Finally, to combine the prediction, bagging (boot-strap aggregating) was implemented, which used “majority voting”. The performance of the model was optimized using early stopping, dropout, and learning rate schedules. This ensemble of transformers processed shape-based information that achieved an f1 score of 42%. Gupta et al. [15] proposed an ensemble model to perform person Re-ID. In this paper, a triple stream of ensemble model was observed. The models used in this model were DeIT as a vision transformer, ResNet-50, and Densenet-101, where the vision transformer interprets the pixel dependency by focusing on every specific patch of images. This model achieved an accuracy of 90.05% and 80.45% on two separate datasets, respectively.

In another study, Ullah et al. [16] proposed a stacked model in which a convolutional neural network was stacked upon a vision transformer. The whole model was then deployed to detect anomalies in video surveillance, where the convolutional neural network detected spatial features and the vision transformer detected long-term temporal relations, which later extracted a spatiotemporal feature. The proposed model achieved an accuracy of 94.6%, 98.4%, and 89.6% in SanghiTech, UCUD Ped2, and CUHK avenue datasets, respectively. In another scholarly inquiry, Ullah et al. [17] implemented a Vision Transformer Anomaly Recognition (ViT-ARN) framework to identify and categorize anomalies in a surveillance camera. This framework had two phases; in the first phase, the anomalies were identified using a tailored, compact, and single-class deep neural network. In the second stage, the anomaly was categorized based on the feature extracted by the vision transformer, which was improved using a bottleneck attention mechanism to improve representation. This ViT-ARN was trained using a total of 858 and 1600 videos from two datasets and was evaluated based on two datasets- LAD-2000 and UCF- Crime datasets where the proposed framework outperformed other state-of-the-art approaches with an increased accuracy of 10.14% and 3% in these two datasets, respectively. In a separate study, Yao et al. [18] proposed a fusion of transformers and CNN for salient object detection (SOD) where the transformer captured the long-distance pixel relationship, and later, a CNN was applied, which extracted the fine-grained local details. This incorporation resolved the problem of using a CNN-based network and showed equal effectivity for both RGB and RGB-D (RGB and depth) SOD. In a different study, Yang et al. [19] presented a novel approach designed for Hyperspectral Image Classification where classification of each pixel was necessary. However, CNN could interpret those local regions quite efficiently but failed to capture the global meaning. On the other hand, the transformer could interpret the global meaning of an image but failed to capture the local region correlation. For this reason, they proposed a fusion of CNN and transformer known as FusionNet, which incorporates the strength of CNN and transformer together.

In a separate study, Nanni et al. [20] demonstrated how the combined power of CNN and transformer could generate a robust performance in segmentation. In this specific research, the authors combined DeepLabv3+, HarDNet-MSEG, and Pyramid Vision Transformers, which resulted in a dice score of 0.875. In another research, Zhang et al. [21] implemented a novel architecture TransFuse which was designed for both 2D and 3D medical image segmentation. In this architecture, the researcher introduced a new fusion technique called “BiFusion” and achieved an accuracy of 94.4% in the ISIC dataset. In another scholarly inquiry, Wang et al. [22] tried to solve the limitation of the U-Net framework in medical segmentation as it could not learn global information. They incorporated the capability of the transformer and CNN to make a new framework known as O-Net, where in the encoder and decoder part, CNN, and swin transformer were used and achieved an accuracy of 80.61%.

All the above-mentioned works generally focused on either convolution or transformer-based classification models or an ensemble of transformer and convolution-based classification models. In the case of ensemble models, the importance of extracted features from every model does not obtain the proper significance because, at the decision level, the class is already classified by a CNN or transformer network. On the other hand, in stacking, the image goes through the model sequentially, which means the extracted feature from CNN or transformer goes through another model where the feature extraction process continues. In this way, the extracted feature is changed by the latter model, which is why the extracted feature in every model does not have the same significance in the final classification. Concerning these findings [12,13,14,15,16,17,18,19,20,21,22], instead of ensembling or stacking the models, this work proposes a hybrid feature extraction method by fusing conventional pre-trained CNN models such as Inception-V3 and ResNet-50 and a transformer model. In this framework, the individual model extracts the feature individually, and later, the extracted features become fused. In this arrangement, extracted features from every individual model obtain the same significance, which provides the framework with superior performance. This triple-stream Conv-ViT consists of three individual models- Inception-V3, ResNet-50, and Vision Transformer. Inception-V3 and ResNet-50 are CNN-assisted networks where convolution is performed for feature extraction. The network uses kernels to extract texture information by correlating between nearby pixels. The Inception-V3 is a large convolutional network that works solely based on convolution. The main advantage of inception-V3 is the usage of several filters, i.e., 277 filters, to detect deep texture features by building correlations between consecutive and nearby pixels [23]. Though Inception-V3 is computationally efficient, it is a very large and complex model which is suspected of vanishing gradient problems. For this reason, the ResNet-50 model is fused to resolve the vanishing gradient problem and extract deep features by building residual connections to nearby pixels. The residual function helps to optimize parameters that enable this model to avoid vanishing or exploding gradients [24]. On the contrary, the vision transformer is focused on building long-distance pixel relationships rather than nearby pixel relationships resulting in shape-based features [25]. Consequently, a transformer model is fused with the Inception-V3 and ResNet-50 models to generate a shape-based texture feature for processing through the deep neural network, which provides the classifier an upper hand in final detection. The major contribution of this research includes:

- Building a three-stream fusion model called Conv-ViT for retinal disease detection by concerning both texture and feature-based information of each class of retinal images.
- Using pre-trained models such as Inception-V3 and ResNet-50, as well as the transformer model, results in a hybrid feature followed by a DNN model to improve model detection performance.
- Conducting laborious experiments for performance analysis, including quantitative, qualitative, and ablation, to signify the model’s effectiveness.

The rest of the paper is organized as follows: Section 2 contains a detailed description of the materials and methods of the proposed Conv-ViT framework with a detailed analysis of all the models. Section 3 represents the performance evaluation of the proposed system, including quantitative study, qualitative study, ablation study, and comparison with previous work in this field. Section 4 and Section 5 contain the discussion and conclusion, respectively.

## 2. Materials and Methods

In this section, a detailed explanation of the working principle of the proposed Conv-ViT network is presented to classify the retinal disease into four classes from the OCT image database. In this approach for feature extraction, a triple stream network is employed, including Inception-V3 and ResNet-50 and vision transformer. The triple-stream network representation of the retinal database assists in identifying four retinal classes after passing through a deep neural network.

### 2.1. Conv-ViT Framework

**Figure 1:**
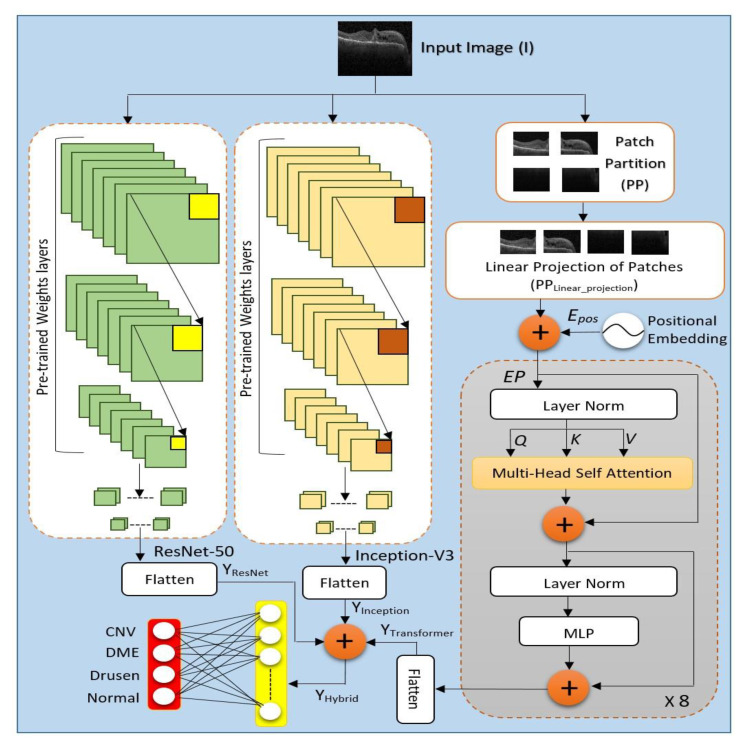
Hybrid Feature Extractor Conv-ViT Framework combines the feature extraction capability of Inception-V3, ResNet-50, and Vision Transformer.

The network architecture of Con-ViT is the fusion of three stream feature extraction models, as illustrated in Figure 1, where the two pre-trained models, including Inception-V3 and ResNet 50, worked along with an attention-based transformer model. This triple-stream network configuration provides robustness to extract hybrid features for final classification through a deep neural network classifier. Moreover, in the following sub-sections of this article, each of the individual models is discussed in part.

#### 2.1.1. Inception-V3

**Figure 2:**
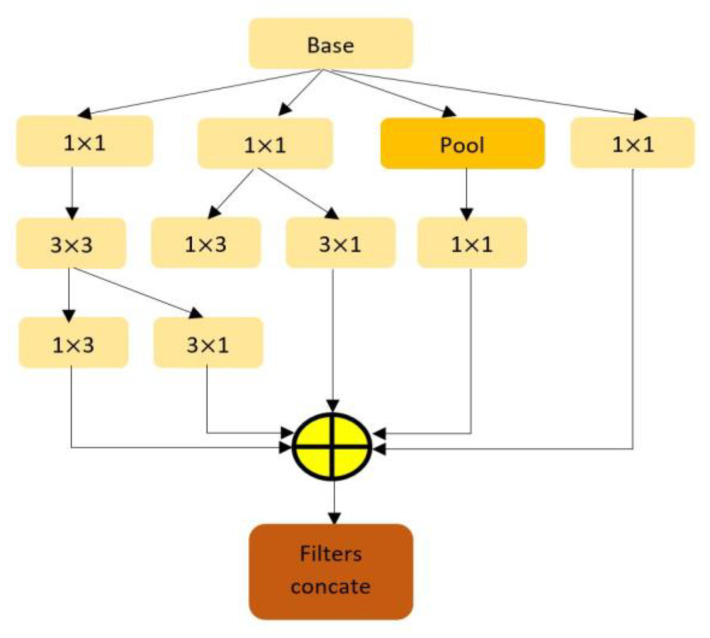
Factorization in Inception Architecture shows how the computation cost is reduced without degrading the performance by using multiple small filters instead of a single large one.

In the initial feature extraction method, the Inception-V3 is selected because of its ability to extract high-level features with several variations of the filters having 277 along with an effective combination of different types of convolution operation. Moreover, the structure of Inception-V3 presents the feature of dimensionality reduction without compromising the model efficiency by using two (3 × 3) layers instead of one (5 × 5) convolution layer because with the same number of filters a (5 × 5) convolution is 25/9 = 2.78 times more computationally expensive than a (3 × 3) convolutions. In this way, using two (3 × 3) convolutions, a total gain of 28% is possible [23]. Additionally, factorizing larger convolution as depicted in Figure 2, the implication of asymmetric convolution results in further time reduction. In addition, instead of (3 × 3) convolutions, a (1 × 3) convolution layer is followed by a (3 × 1) convolution layer reduces the computational cost. For the (3 × 3) convolution layer, the total number of parameters found is 9. On the other hand, a (1 × 3) convolution layer followed by a (1 × 3) convolution layer results in a total number of parameters of (3 × 1) + (1 × 3) = 6, which also reduces the total number of parameters by around 33%. Concerning the computational cost, the Inception-V3 architecture additionally utilized an efficient grid reduction technique to support issues of conventional pre-trained models. As the general pre-trained model uses max pooling followed by a convolution layer that is too greedy. On the other hand, the utilization of max-pooling after the convolution layer is too expensive. To address the issues, Inception-V3 performed the efficient grid reduction using separate convolution and pooling operations followed by the final concatenation.

For the feature extraction task, initially, the images, *I* with size (84 × 84 × 3) are fed to the Inception-V3 excluding the auxiliary classifier component that results in texture features, *Inception_feature_texture_* from the last concatenate layer (mixed10) and the conversion of the texture feature into a 1D vector is performed using a flattened layer that generated an output of *Y_Inception_* as presented in Equations (1) and (2):

(1) $$Inception_{feature\_texture}=Inception_{mixed10\_layer\left(I\right)}$$

(2) $$Y_{Inception}=flatten\left(Inception_{feature\_texture}\right)$$

#### 2.1.2. ResNet-50

**Figure 3:**
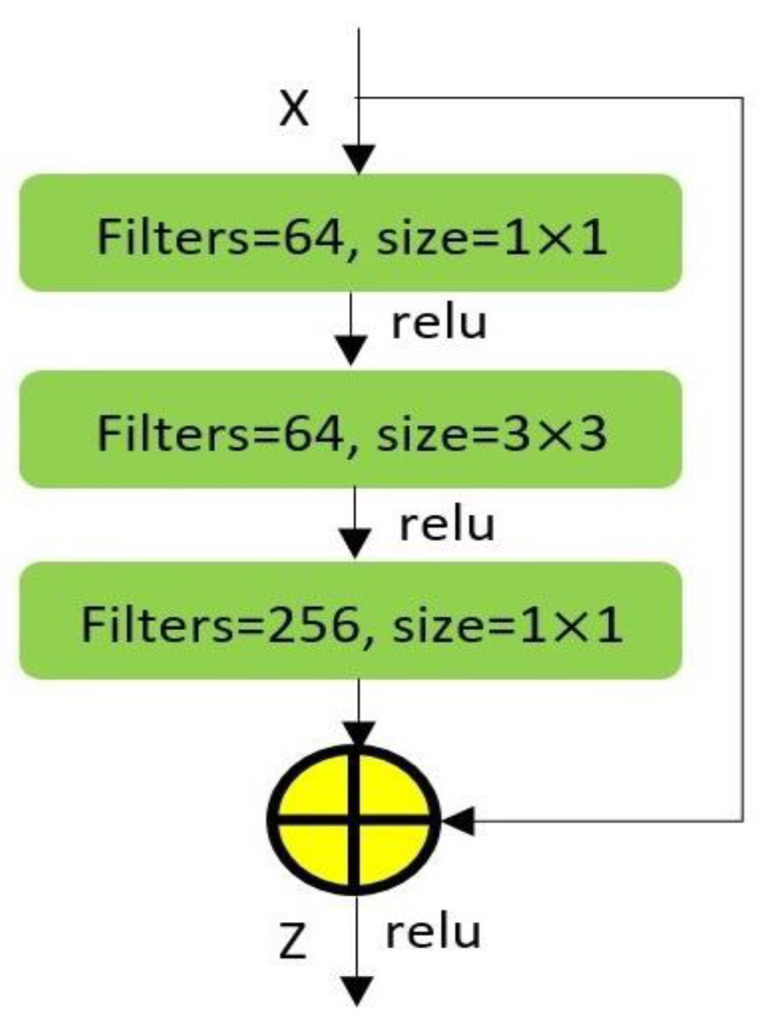
Three-layer Residual function Block; the basic building block of ResNet-50. This residual function block is stacked on top of each other in ResNet-50 architecture.

In addition to high-level feature extraction from the Inception-V3 model, the ResNet-50 is employed to focus on low-level features as well as using the residual connections in the architecture. During convergence, the Inception-V3 network performance gets saturated and degrades a bit after that [26]. To tackle these problems, this work introduces ResNet-50 for the feature extraction process. In the ResNet-50 architecture, there are 50 layers within five blocks. For each of these blocks, the residual function, F contains three convolution layers as shown in Figure 3 with dimensions (1 × 1), (3 × 3), and (1 × 1). The output, *Z,* of this block is calculated by adding the input, *x,* with the residual function, *F,* as represented in Equation (3). Where the residual function *F* updates the input *x* with a weight matrix *W_i_* of three consecutive convolution layers. During feature extraction, the input image, *I,* with shape (84 × 84 × 3), is fed to the ResNet-50 network, and the output of the *conv5_block3_out layer*, *ResNet_conv5_block3_out_layer_* is used for final classification. The texture feature, *ResNet_feature_texture_* is then converted using a flattened layer to generate a 1D vector output of *Y_ResNet_*.

(3) $$Z=F\left(x,W_{i}\right)+x$$

(4) $$ResNet_{feature\_texture}=ResNet_{conv5\_block3\_out\_layer\left(I\right)}$$

(5) $$Y_{ResNet}=flatten\left(ResNet_{feature\_texture}\right)$$

#### 2.1.3. Vision Transformer

In addition to the kernel-based texture features, which have been extracted from the CNN-based model, a third-stream model called vision transformer (ViT) has been utilized in the Con-ViT network. The ViT works based on the attention mechanism by developing relationships between nearby pixels as well as long-distance pixels. To perform the tasks of attention mechanism, the input image is first divided into small patches. This process is analogous to a convolution layer using a kernel where the output is a 4D matrix that is indexed by batch, and the other three dimensions are row, column, and depth. Thereby, the image *I* ∈ ${\mathbb{R}}^{H\times W\times C}$ is reshaped into *PP* ∈ ${\mathbb{R}}^{N\times P^{2}\times C}$ where *H* and *W* are the width and height of the image, and *C* represents the number of channels. On the other hand, *N* is the number of patches calculated as

(6) $$N=\frac{H\times W}{P^{2}}$$

where *P* is the patch size. The image size is (78 × 78) and the patch size is (6 × 6) as shown in Table 1. From the image and patch size, the number of patches is calculated: ($\frac{H\times W}{P^{2}})=\frac{78\times 78}{{\left(6\right)}^{2}}=169$. After patch partition, the raw image (*I*) is converted into a 2D matrix, *PP* and is linearly projected into a 1D embedding vector, $PP_{Linear\_Projection}$ with a dimension of 64:

(7) $$PP=\left[\begin{array}{cccc} \left[I_{1}\right] & \left[I_{2}\right] & \ldots \; & \left[I_{13}\right]\\ \left[I_{14}\right] & \left[I_{15}\right] & \ldots & \left[I_{26}\right]\\ \ldots & \ldots & \ldots & \ldots \\ \left[I_{157}\right] & \left[I_{158}\right] & \ldots & \left[I_{169}\right] \end{array}\right]$$

(8) $$PP_{Linear\_Projection}=\left[\begin{array}{cccc} {}[I_{1}^{1} & I_{1}^{2} & \ldots \; & I_{1}^{64}]\\ {}[I_{2}^{1} & I_{2}^{2} & \ldots & I_{2}^{64}]\\ \ldots & \ldots & \ldots & \ldots \\ {}[I_{169}^{1} & I_{169}^{2} & \ldots & I_{169}^{64}] \end{array}\right]$$

As the performance of the transformer is computationally expensive, the patches are embedded with a positional embedding where the image patches are grouped into smaller groups and further applied to larger image sizes [27]. The position embedding, $E_{POS}$ is performed based on the mixing of sine and cosine functions of different frequencies [28]. If the patch is in an odd position, use the function of cosine, and on the contrary, the even position patch is embedded using the sine function. Here, *pos* refers to position, whereas *i* refers to dimension, and the whole positional embedding is encoded in different positions of this sinusoid. Again, *d* is the maximum length of the patch group. Then, the linear projected patch is concatenated with a positional embedding which later produces an embedded patch.

(9) $$E_{POS}=\left\{\begin{array}{c} \sin(\frac{pos}{1000^{\frac{2i}{d}}}),\;i\;\mathrm{is}\;\mathrm{even}\\ \cos\left(\frac{pos}{1000^{\frac{2i}{d}}}\right),\;i\;\mathrm{is}\;\mathrm{odd} \end{array}\right.$$

(10) $$EP=concatenate\left(PP_{Linear_{Projection}},E_{POS}\right)$$

After the linear projection and positional embedding, the embedded patch is passed on to the encoded block. The encoder is a repetition of eight similar blocks, each having a combination of six layers, including a NORMALization layer followed by a multi-head self-attention (MSA) layer and multi-layer perceptron (MLP). At first, the input of the encoder block, such as *EP,* is concatenated with the output of MSA. Later, the output passes onto a NORMALization layer, and MLP has a dense dropout layer. Finally, a skip connection from the input gets concatenated with this attention output which increases the impact of position as the next layer is provided with the original embedded patch. The calculation of attention is performed using three embedding matrices, such as *key K*, *query Q*, and *value V*, where the matrices are calculated using weight matrices *W_Q_*, *W_K,_* and *W_V_* by using the following equations:

(11) $$Query,\;Q=EP.W_{Q}$$

(12) $$Key,\;K=EP.W_{K}$$

(13) $$Value,\;V=EP.W_{V}$$

where *EP* is the embedded patch, and the weight matrices are- *W_Q_, W_K_, W_V_* ∈ ${\mathbb{R}}^{d_{model}\times d_{k}}$ The single attention function called head is performed using the following equation that is parallelly executed multiple times in the *MHA* layer, where the attention is calculated using Equation (14). Where *d_k_* is a dot-scaled product that prevents the attention value from exploding. This attention value works as a scoring function that represents the correlation between two image patches. In the proposed framework, the multi-head attention (*MHA*) has four heads, so the representation is as follows:

(14) $$Attention,\;\left(Q,\;K,\;V\right)=softmax\left(\frac{QK^{T}}{\sqrt{d_{k}}}\right)V$$

(15) $$MHA=Attention,\;{\left(Q,\;K,\;V\right)}_{\times 4}$$

In the later layers, the multi-layer perceptron is used, which uses a dense layer with Gaussian error linear unit (*GELU*) activation, which provides non-linearity in this process and where ϕ is the cumulative distribution of Gaussian distribution. Finally, the output of the layer is taken out, which is *Transformer_feature_shape_*, and later, we flatten it as per the following equations:

(16) $$GELU\left(x\right)=xP\left(X\leq x\right)=x\phi \;\left(x\right)$$

(17) $$Transformer_{feature\_shape}=GELU\left(MHA\right)$$

(18) $$Y_{Transformer}=flatten\left(Transformer_{featur\_shape}\right)$$

Then, the *Y_Transformer_* is passed onto the deep neural network classifier. Moreover, the required optimum parameters for the transformer feature extractor of the Conv-ViT network are summarized in Table 1. Where the value of the parameters was first anticipated by considering the complexity and size of the dataset and later was tuned to make them more optimized for which the model’s performance on the validation dataset was considered.

#### 2.1.4. Deep Neural Network (DNN) Classifier

**Figure 4:**
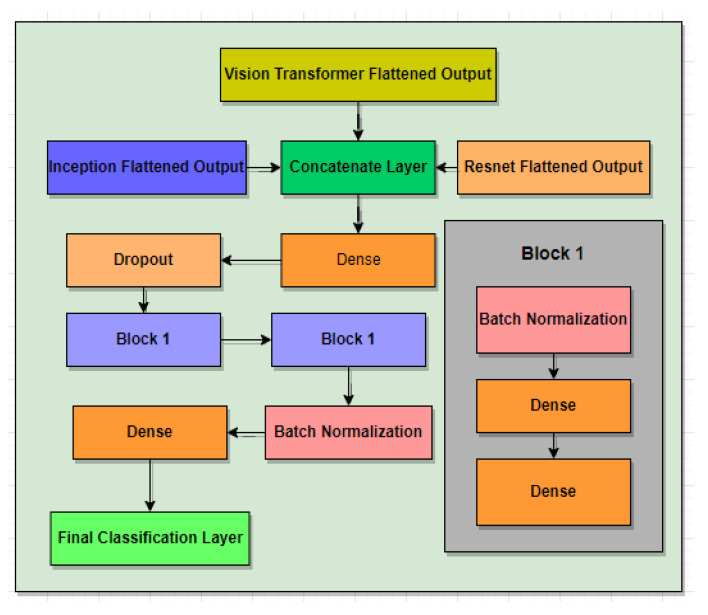
Functional structure of a deep neural network classifier. This DNN classifier predicts the class from the extracted feature.

Figure 4 shows the functional structure of the deep neural network classifier that predicts a class from the extracted feature. After taking the features from individual models, the concatenation is performed on ConvNet-produced texture-based features and vision transformer-produced shape-based features. Consequently, the *concatenation* layer produces a single one-dimensional hybrid feature vector, a *Y_Hybrid_* feature comprising texture and shape-based features. After that, the DNN classifier is employed for the final classification, where the *Y_Hybrid_* passes through *dense* and *dropout* layers to produce *Y*_1_. Then, the *Y*_1_ is followed by two repetitive blocks named *Block*1 that contain three layers of one *batch normalization* and two *dense* layers. Finally, a *dense* layer is used, followed by a *softmax* layer for retinal disease classification.

(19) $$Y_{Hybrid}=concatenate\left(Y_{Inception},Y_{Resnet},Y_{Transformer}\right)$$

(20) $$Y_{1}=dropout\left(dense\left(Y_{Hybrid}\right)\right)\;$$

(21) $$Block1=f\left(batchnormalization,dense,dense\right)$$

(22) $$Y_{2}=Block1\left(Y_{1}\right)$$

(23) $$Y_{prediction}=softmax\left(Y_{2}\right)$$

### 2.2. Hyperparameter Settings

In the case of model training, the hyperparameters are selected and tuned for optimizing the model prediction. The selected model hyperparameter and tuning technique are mentioned in Table 2. During the training, the categorical cross entropy loss function is employed, and the weights are updated using the Adam optimizer. The Adam optimizer reduces the error by updating weights that result in optimum model performance. The proposed model is trained for twenty iterations, and the learning rate is optimized using a learning rate scheduler.

The experiment of developed Conv-ViT model is performed on the Google Colab environment provided by google, located in Mountain View, California, USA with Python 3 for both training and testing. The platform has a GPU facility to accelerate the training process faster by enhancing the computational speed. For preprocessing, the NumPy is used for model evaluation. In addition, Keras and Tensorflow 2.9.2 framework, also developed by google, is employed for model implementation and visualization.

### 2.3. Dataset

The dataset used for training and evaluation is a public dataset collected from Mendeley [29]. The images are collected as a part of a routine checkup, and in the dataset, a foveal cut of the original image is used [7]. The original distribution of the validation and test set is changed to reflect the original distribution of the training set. In this distribution, the sample ratio of each class is kept constant in the train, test, and validation set, as this helps the model to simulate a result that will approximate the real-world scenario more accurately [30]. The number of sample images in each class is given in Table 3. Table 3 represents the dataset containing a total of 109,309 images divided into 4 classes. The dataset used here is highly imbalanced, and the percentage of training, validation, and test set are 90%, 5%, and 5%.

### 2.4. Evaluation Metrics

For evaluation purposes, *Accuracy*, *Precision*, *Recall*, and *F*1 *score* are used as evaluation metrics. Now, *precision*, *recall*, and *F*1 *score* are given more emphasis rather than *accuracy* because the dataset is imbalanced, which can be observed from Table 3. The representation of the matrices is given below.

(24) $$Accuracy=\frac{TP+TN}{TP+TN+FP+FN}$$

(25) $$Precision=\frac{TP}{TP+FP}$$

(26) $$Recall=\frac{TP}{TP+FP}$$

(27) $$F1\;Score=2\times \left(\frac{Precision\times Recall}{Precision+Recall}\right)$$

where *TP*, *TN*, *FP*, and *FN* represent true positive, true negative, false positive, and false negative, respectively. The *accuracy* indicates how the model detects a class as positive among all other samples. On the contrary, *precision* represents how exact or precise a model is in detecting a class as positive among all the samples detected as positive, which is the ratio of true positive and the sum of true positive and false positive. Moreover, *recall* is the ratio of true positives and the sum of true positives and false negatives, indicating how the model detects a class among all the classes detected. The *F*1 *score* is the weighted average of *precision* and *recall*.

## 3. Results and Analysis

**Figure 5:**
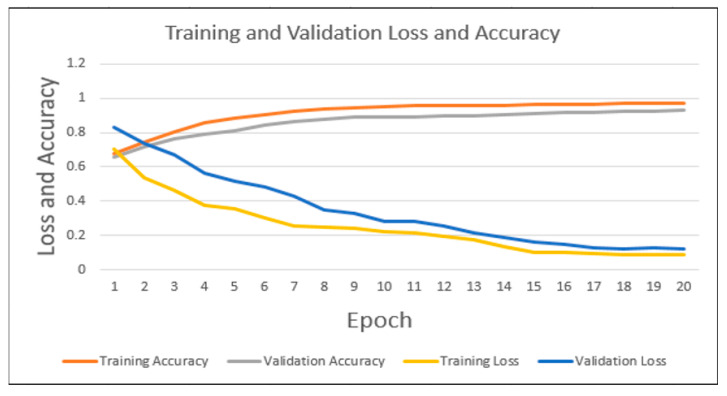
Loss and accuracy graph for train and validation set. The curve shows how the loss and accuracy in the training and validation set are changing with respect to epoch.

After training the model for 20 epochs due to GPU constrained, the test set is used to evaluate this model. The model is trained for approximately 3 h, which means each iteration takes about 10 min to complete. Figure 5 represents the variation of loss and accuracy in terms of epoch for the train and validation set. Overall, the accuracy curve for the training and validation set showed an upward trend, while the loss curve for the training and validation set displayed a downward trend. First, the training loss and validation loss are decreasing in nature, and the training loss and validation loss become almost constant in nature after 14 epochs. The variation in this duration of the first 14 epochs is 0.70~0.13 and 0.83~0.18 for training loss and validation loss, respectively. In terms of training accuracy and validation accuracy, the curves displayed an increasing trend till 14 epochs, and after that, the curves almost plateaued. In this duration of 14 epochs, the training accuracy varies from 67.51% to 96.13%, and validation accuracy varies from 65.82% to 90.64%. After completing the training, the training and validation accuracy is 97.45% and 92.89%, respectively. On the other hand, the training loss is 0.0882, and the validation loss is 0.1232. In conclusion, Figure 5 displayed how the model trained and improved in terms of accuracy and loss during the first 14 epochs, and beyond 14 epochs, the accuracy and loss curves plateaued, which indicates the model had converged after this certain period.

### 3.1. Quantitative Analysis

**Figure 6:**
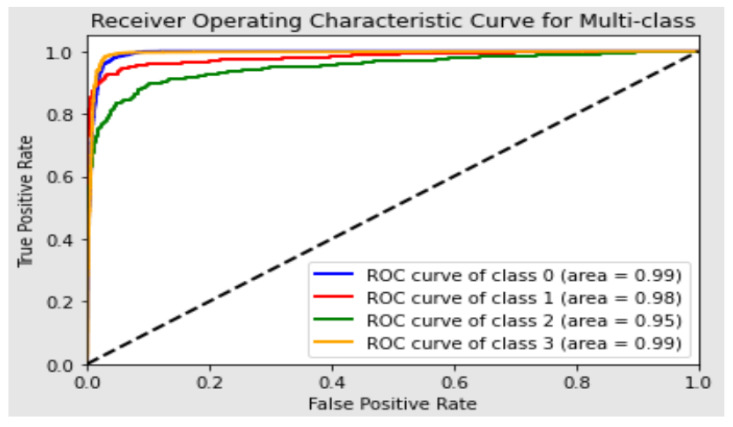
The AUC curves evaluate the model’s ability to classify between these four classes. Each curve represents a separate curve, and the area under the curve represents how a class is differentiated from the other three.

In quantitative analysis, the performance of the model is evaluated and analyzed using the quantitative value of four metrics. The model performance is evaluated using the test set. In terms of the test set, the model shows an overall accuracy of 94.46%. The precision and recall of the model are found to be 0.9447 and 0.9425, respectively. The F1 score is achieved at 0.9436. In addition, the class-wise performance of the Conv-ViT model is evaluated, as summarized in Table 4, due to highly imbalanced dataset distribution. The highest F1 score of 0.98 is found for the NORMAL class, and the lowest score of 0.78 is obtained for the DRUSEN class. The recall of the DRUSEN class is lower than the precision, which indicates that the false negative in the DRUSEN class reduced the model’s performance. On the other hand, the DME and CNV classes have an F1 score of 0.89 and 0.94, respectively. The weighted average of the F1 score is 0.94, whereas the macro average is 0.89. From Table 4, it can be observed that the highest precision, recall, and F1 score are found as 0.98, 0.99, and 0.98, respectively. Moreover, Figure 6 shows the AUC curve for each class with an overall accuracy of 0.98. In this case, class 0, class 1, class 2, and class 3 refer to CNV, DME, DRUSEN, and NORMAL, respectively. The figure shows that the lowest AUC score is 0.95 for the DRUSEN class. On the other hand, it is 0.99 for CNV and NORMAL classes. The AUC score is 0.98 for the DME class. The overall AUC score of 0.98 indicates that the model has a probability of 0.98 in the case of classifying a randomly chosen sample as positive higher than a randomly chosen negative instance.

### 3.2. Qualitative Analysis

The qualitative performance of the proposed Conv-ViT model is evaluated using sample images where the error is calculated based on the correct or wrong prediction of the model. For this evaluation purpose, an image from each class is chosen. After that, the image is given to the models, and then the error is analyzed. The qualitative analysis is performed using seven different models where all the models are trained using the same model parameter and tuning technique. All the models have their unique feature extraction techniques. Table 5 contains all the predictions that are made by these models. The first sample is from CNV class which is correctly predicted by all the models. That means this class is not dependent on the combination of shape or texture-based features. Any of these standalone feature extractors can classify this class. On the contrary, only four of the models can classify the DME class. Analyzing the models, the DME class is predicted correctly when the shape-based feature extractor, such as ViT, is present. In the case of the DRUSEN class, only the proposed mode is predicted correctly because of extracting a combination of three types of features: specific texture-based feature by Inception-V3, generalized texture-based feature by ResNet-50, and shape-based feature by ViT. Despite having lower samples in this class, the proposed model is capable of identifying the class correctly. Finally, in the case of the NORMAL class sample, only ViT produces a wrong prediction. The ViT-produced shape-based feature is not adequate to predict the NORMAL class. Therefore, all other models associated with a ConvNet or a combination of ConvNet and ViT can produce either texture-based or hybrid features that are required to predict the NORMAL class correctly.

Among the seven models, Inception-V3, ResNet-50, and ViT are used as standalone feature extractors. All these models predicted two of the classes properly. The Inception-V3 and ResNet-50 Predicted DME and DRUSEN as CNV and NORMAL, respectively. On the other hand, in the case of ViT, the false negative is DRUSEN and NORMAL, which are predicted as NORMAL and DME. The combination of Inception-V3 and ResNet-50 feature extractors can extract two types of texture-based features. This feature extractor cannot predict DME and DRUSEN class. This class needs a shape-based feature that cannot be extracted using the combination of Inception-V3 and ResNet-50. The ViT is used with Inception-V3, which cannot predict the DRUSEN class. However, ViT with ResNet-50 has produced a false negative for the DRUSEN class. On the other hand, the proposed model predicts all the classes correctly.

### 3.3. Ablation Study

To justify the effectiveness of the self-attention component in the proposed Conv-ViT framework, several experiments are performed on different networks with and without utilizing the self-attention component. Table 6 represents the impact of self-attention on the model’s performance for different classes of OCT images. In the case of CNV class, Inception-V3 performs better without self-attention, where removing the self-attention increases the accuracy from 92.41% to 93.32%, and the F1 score increases from 0.92 to 0.93. On the contrary, self-attention on ResNet-50 makes this model more efficient by increasing the accuracy by 2.35%, and the F1 score is increased from 0.91 to 0.92. In terms of the combination of Inception-V3 and ResNet-50, the accuracy and F1 score decreases because of using self-attention. The accuracy decreases from 94.87% to 92.09%, and the F1 score decreases from 0.93 to 0.91. Again, the combination of the three networks using sum fusion produced an accuracy and F1 score of 94.83% and 0.93 when self-attention was applied on all of the models and 94.27% and 0.93 without self-attention in the convolution-based network. For the proposed framework, self-attention increases the accuracy from 94.55% to 96.74%, and the F1 score rises to 0.95 from 0.94. For the DME class, Inception-V3 performs better without self-attention, where the accuracy increases from 0.72 to 0.75. Again, the ResNet-50 performs better with self-attention, where the accuracy increases from 71.03% to 69.83%, and the F1 score increases from 0.72 to 0.71. Moreover, the combination of Inception-V3 and ResNet-50 works better with self-attention. The accuracy and F1 score increased by 1.55% and 0.04, respectively. In this class, the accuracy and F1 score both increased by 2.27% and 0.01, which was 85.78% and 0.85, respectively, when self-attention was only used in the vision transformer. The proposed framework performance decreases when self-attention is used for the individual ConvNet model. The accuracy decreases from 90.07% to 87.07%, and the F1 score decreases from 0.89 to 0.87. On the contrary, for the DRUSEN class, the Inception-V3 extractor works better without self-attention, where the F1 score increases from 0.48 to 0.49. ResNet-50 works better with self-attention, where the accuracy increases from 33.86% to 37.92%, and the F1 score increases from 0.41 to 0.39. The self-attention in the combination of Inception-V3 and ResNet-50 increases the accuracy from 37.02% to 36.52%. Furthermore, the sum fusion of the three models performed better without self-attention, where the accuracy increased from 58.61% to 60.76%, and the F1 score rose to 0.60 from 0.56. The Conv-ViT framework performs better without self-attention, where the accuracy decreases by 6.19%, and the F1 score also decreases by 0.03. In the NORMAL class, the accuracy of Inception-V3 increases to 96.34%, and the F1 score increases to 0.93. The ResNet-50 with self-attention shows a minor impact though there is a slight increase of 0.82% in accuracy when self-attention is applied; the F1 score remains constant. The combination of Inception-V3 and ResNet-50 works better with self-attention, where the accuracy increases to 95.60% from 94.05%, and the F1 score Increases to 0.93 from 0.92. While the F1 score for sum fusion of Inception V3, ResNet-50, and vision transformer remained constant with and without self-attention, the accuracy had a slight increase from 96.12% to 96.38% when self-attention was not applied. The Conv-ViT framework works better without self-attention with an increment of the accuracy of 1.34% though the F1 score remains constant. However, except for CNV class, the proposed Conv-ViT framework without self-attention in Inception-V3 and ResNet-50 outperforms all other models. Moreover, our proposed framework, where the fusion was performed using concatenation, achieved a higher accuracy and F1 score in every class than the sum fusion of these three models, which indicates that combining features with the concatenation method increased the model’s capability to interpret the complex interaction of features than fusing them using sum rule.

The observation from the ablation study can also verify the findings of the qualitative study. From qualitative analysis, it is observed that DME is correctly observed when there is a shape-based feature extractor. In Table 6, for the DME class, there is a significant increase in the F1 score from 0.76 to 0.89 when the vision transformer is used with Inception-V3 and ResNet-50. On the other hand, for the DRUSEN class, the F1 score is low compared to other classes. However, the proposed model has a comparatively high F1 score of 0.77, whereas, in the case of other models, the highest F1 score is 0.49. In the qualitative study, only the proposed Conv-ViT correctly predicted this class. Moreover, in the case of CNV and NORMAL classes, the performance of the models is comparatively constant for other models. For CNV class, the performance of the models varied from 0.91 to 0.95, and for the NORMAL class, the performance of the models is in the range of 0.91 to 0.97, which supports the qualitative analysis as the sample for CNV and NORMAL class is classified correctly by most of the models.

### 3.4. Analysis of Significance of Feature Level Concatenation

Table 7 evaluates the performance of two strategies, feature-level concatenation and decision-level concatenation, implemented within the Conv-ViT framework. In the case of decision-level concatenation majority voting technique was used. The feature level concatenation outperformed the decision level concatenation with an accuracy of 94.46%, whereas it was 87.36% for decision level concatenation. The value of precision, recall, and F1 score were persistently higher in feature level concatenation with values of 0.94, 0.94, and 0.94, respectively. On the other hand, the precision, recall, and F1-score for decision-level concatenation were 0.87, 0.86, and 0.86, respectively. This analysis implies that combining features at the feature level can provide better performance than combining decisions at the decision level.

### 3.5. Complexity Analysis

Table 8 analyzes the computational complexity of the proposed hybrid Conv-ViT framework over individual models in terms of multiply-accumulate (MAC) operation. The number of parameters in the MAC unit for the proposed Con-ViT framework is found to be around 93 M, which is a bit larger compared to individual models, including Inception-V3, ResNet-50, and Vision Transformer to capture complex patterns of the retinal images.

## 4. Discussion

In pursuit of observing the generalization capability of the proposed framework, as presented in Table 9, the model is tested on another dataset called the optical coherence tomography image database (OCTID). The dataset is collected from the work of *P*. Gholami et al. [31]. This dataset consists of two classes, including AMD and NORMAL. The accuracy and F1 score are found to be 92.37% and 0.92, respectively, on this dataset which closely approximates the performance on the Mendeley [29] dataset. Therefore, this is evidence of the proposed model’s good generalization on retinal disease classification.

A comparative analysis of the proposed framework performance with the existing state-of-the-art models on Mendeley [29] and OCTID [31] datasets is performed as presented in Table 10. The results are regenerated on this dataset for all the existing models to compare performance superiorly with the proposed framework. Among the model examined, the proposed Conv-ViT framework demonstrates the highest accuracy of 94.46%. This indicates the robustness of the model compared to other state-of-the-art techniques. The proposed framework outperforms other state-of-the-art models, including Inception-V3 [23], ResNet-50 [26], Vision Transformer [27], VGG-16 with initialized weight [32], and iterative fusion convolutional neural network [33]. While the other models performed significantly better, with an accuracy of more than 80%, the vision transformer performed poorly, with an accuracy of 65.95%. The experimental results provide insight into the limited efficacy of a shape-based extractor, such as the vision transformer, in robustly detecting different types of AMD, while it also proves enough evidence about the performance enhancement achieved by incorporating a texture-based feature extractor. This analysis signifies the excellence of the proposed framework specifying the prospect of the Conv-ViT framework in classifying different types of age-related macular degeneration. As a part of a further evaluation of the performance of the Conv-ViT framework on the OCTID dataset was also compared with other state of the other art method. Table 10 demonstrated that our proposed framework had the highest accuracy of 92.37%, whereas Inception V3 achieved second place with an accuracy of 85.03%. This evaluation illustrates that the proposed framework can produce robust and significant results irrespective of the dataset being used.

## 5. Conclusions

In this paper, a hybrid feature extraction method is proposed with the inclusion of Inception-V3, ResNet-50, and ViT model where Inception-V3 and ResNet-50 extract specialized and generalized texture-based features and on the other hand, attention-assisted ViT network extract shape-based feature. The combination of these three types of features makes the Conv-ViT framework flavorsome in the detection of three types of age-related macular degeneration and separates them from NORMAL OCT images. With the help of extracting triple stream features from OCT images, this model outperformed some notable work in the field of macular degeneration grading. Despite outperforming the proposed triple stream model over the single stream models, the higher computational complexity should have been the concern for the practical feasibility of the model. Thereby, as an extension of the work, the time complexity could be reduced in the future. In addition, concerning the robustness of the model, a high-resolution image can be processed with GPU availability for classifying different types of age-related macular degeneration.

## Figures and Tables

**Table 1:** Selected optimum parameters of the vision transformer.

Parameter	Value
Image Size	78
Patch Size	6
Number of Patch	169
Projection Dimension	64
Number of Heads	4
Transformer Unit	(128, 64)
Number of Encoder	8
MLP Head Unit	(2048, 1024)

**Table 2:** Selected hyperparameters value for the proposed Conv-ViT model.

Hyperparameter	Value
Loss Function	Categorical Crossentropy
Number of Epoch	20
Optimizer	Adam
Batch Size	64
Tuning Technique	Learning Rate Scheduler

**Table 3:** Distribution of dataset for each class in train, test, and validation.

Class	Train	Validation	Test	Total
CNV	33,711	1872	1872	37,455
DME	10,438	580	580	11,598
DRUSEN	7980	443	443	8866
NORMAL	46,250	2570	2570	51,390
TOTAL	98,379	5465	5465	109,309

**Table 4:** Class-wise performance analysis of proposed Conv-ViT network.

Class	Precision	Recall	F1 Score
CNV	0.94	0.95	0.94
DME	0.88	0.89	0.89
DRUSEN	0.83	0.72	0.78
NORMAL	0.98	0.99	0.98
Macro Average	0.90	0.89	0.89
Weighted Average	0.94	0.94	0.94

**Table 5:** Prediction of each class from sample images on different model variations. The symbol (✓) and (×) indicates the correct and incorrect prediction, respectively.

Class	Model	Actual Class	Predicted Class
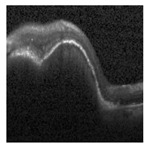	Inception-V3	CNV	CNV (✓)
	ResNet-50		CNV (✓)
	Inception-V3 + ResNet-50		CNV (✓)
	ViT		CNV (✓)
	Inception-V3 + ViT		CNV (✓)
	ResNet-50 + ViT		CNV (✓)
	Inception-V3 + ResNet-50 + ViT (Proposed)		CNV (✓)
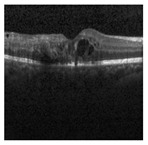	Inception-V3	DME	CNV (×)
	ResNet-50		CNV (×)
	Inception-V3 + ResNet-50		CNV (×)
	ViT		DME (✓)
	Inception-V3 + ViT		DME (✓)
	ResNet-50 + ViT		DME (✓)
	Inception-V3 + ResNet-50 + ViT (Proposed)		DME (✓)
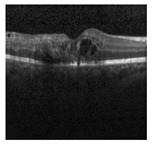	Inception-V3	DRUSEN	NORMAL (×)
	ResNet-50		NORMAL (×)
	Inception-V3 + ResNet-50		CNV (×)
	ViT		NORMAL (×)
	Inception-V3 + ViT		NORMAL (×)
	ResNet-50 + ViT		NORMAL (×)
	Inception-V3 + ResNet-50 + ViT (Proposed)		DRUSEN (✓)
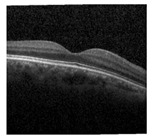	Inception-V3	NORMAL	NORMAL (✓)
	ResNet-50		NORMAL (✓)
	Inception-V3 + ResNet-50		NORMAL (✓)
	ViT		DME (×)
	Inception-V3 + ViT		NORMAL (✓)
	ResNet-50 + ViT		NORMAL (✓)
	Inception-V3 + ResNet-50 + ViT (Proposed)		NORMAL (✓)

**Table 6:** Significance of attention component on proposed Conv-ViT model. The symbol (✓) and (×) means with and without self-attention mechanism respectively. Bold font indicates the result of the proposed framework.

Class	Model	Self-Attention	Accuracy	F1 Score
CNV	Inception	✓	92.41%	0.92
		×	93.32%	0.93
	ResNet	✓	91.88%	0.92
		×	94.23%	0.91
	Inception + ResNet	✓	94.87%	0.93
		×	92.09%	0.91
	Inception + ResNet + ViT (Sum Rule Fusion)	✓	94.83%	0.93
		×	94.27%	0.93
	Inception + ResNet + ViT(Concatenation)	✓	96.74%	0.95
		×	**94.55%**	**0.94**
DME	Inception	✓	72.41%	0.72
		×	71.03%	0.75
	ResNet	✓	71.03%	0.72
		×	69.83%	0.71
	Inception + ResNet	✓	71.21%	0.76
		×	69.66%	0.72
	Inception + ResNet + ViT (Sum Rule Fusion)	✓	83.51%	0.84
		×	85.78%	0.85
	Inception + ResNet + ViT(Concatenation)	✓	87.07%	0.87
		×	**90.07%**	**0.89**
DRUSEN	Inception	✓	42.66%	0.48
		×	41.53%	0.49
	ResNet	✓	37.92%	0.41
		×	33.86%	0.39
	Inception + ResNet	✓	37.02%	0.45
		×	36.57%	0.43
	Inception + ResNet + ViT (Sum Rule Fusion)	✓	58.61%	0.56
		×	60.76%	0.60
	Inception + ResNet + ViT(Concatenation)	✓	69.30%	0.75
		×	**75.49%**	**0.78**
NORMAL	Inception	✓	92.96%	0.92
		×	96.34%	0.93
	ResNet	✓	92.96%	0.91
		×	92.14%	0.91
	Inception + ResNet	✓	95.60%	0.93
		×	94.05%	0.92
	Inception + ResNet + ViT (Sum Rule Fusion)	✓	96.12%	0.95
		×	96.38%	0.95
	Inception + ResNet + ViT(Concatenation)	✓	96.73%	0.97
		×	**98.43%**	**0.98**

**Table 7:** Significance of feature level concatenation over decision level concatenation in Conv-ViT framework.

Performance Matrices	Feature Level Concatenation	Decision Level Concatenation
Accuracy	94.46%	87.38%
Precision	0.94	0.87
Recall	0.94	0.86
F1 Score	0.94	0.86

**Table 8:** The computational complexity of the proposed Con-ViT framework in terms of multiply accumulate (MAC) operation.

Models	Parameters (MAC)
Inception-V3	24,527,268
ResNet-50	43,070,724
Vision Transformer	26,645,252
Conv-ViT	92,919,716

**Table 9:** Performance of proposed framework on Mendeley and OCTID datasets.

Dataset	Predicted Class	Accuracy	F1 Score
Mendeley [29]	CNV DME DRUSEN NORMAL	94.46%	0.94
OCTID [31]	AMD NORMAL	92.37%	0.92

**Table 10:** Comparison of the proposed framework performance with existing state-of-the-art methods on the Mendeley and OCTID Datasets (Bold font indicates the best results).

Reference No.	Model	Accuracy (%)	
		Mendeley Dataset	OCTID Dataset
Szegedy et al. [23]	Inception-V3	88.18	85.03
He et al. [26]	ResNet-50	85.51	80.09
Trockman et al. [27]	Vision Transformer	65.95	61.37
Li et al. [32]	VGG-16 with Initialized Weight	82.53	84.19
Fang et al. [33]	IFCNN	89.56	82.47
Proposed Method	Conv-ViT	**94.46**	**92.37**

## Data Availability

The dataset used for the experiment is publicly available at https://data.mendeley.com/datasets/rscbjbr9sj/3 (accessed on 7 September 2022). The second dataset, which is used to observe the generalization capability of the model, is also available at https://borealisdata.ca/dataverse/OCTID (accessed on 2 February 2023). The necessary code to implement the Conv-ViT framework is available at https://github.com/PramitDutta1999/Conv-ViT-A-Convolution-and-Vision-Transformer-Based-Hybrid-Feature-Extraction-Method (accessed on 31 March 2023).

## References

[1] Ram A., Reyes-Aldasoro C.C. (2020). The Relationship between Fully Connected Layers and Number of Classes for the Analysis of Retinal Images. arXiv.

[2] National Eye Institute Age-Related Macular Degeneration (AMD). https://www.nei.nih.gov/learn-about-eye-health/eye-conditions-and-diseases/age-related-macular-degeneration#section-id-7323.

[3] Ferrara N. (2010). Vascular endothelial growth factor and age-related macular degeneration: From basic science to therapy. Nat. Med..

[4] Varma R., Bressler N.M., Doan Q.V., Danese M.D., Dolan C.M., Gower E.W., Greenberg P.B., Jampol L.M., Kinyoun J.L., Kolomeyer A.M. (2014). Prevalence of and Risk Factors for Diabetic Macular Edema in the United States. JAMA Ophthalmol..

[5] Friedman D.S., O’Colmain B.J., Muñoz B., Tomany S.C., McCarty C., de Jong P.T.V.M., Nemesure B., Mitchell P., Kempen J. (2004). Prevalence of Age-Related Macular Degeneration in the United States. Arch. Ophthalmol..

[6] Wang D., Wang L. (2019). On OCT Image Classification via Deep Learning. IEEE Photonics J..

[7] Kermany D.S., Goldbaum M., Cai W., Valentim C.C.S., Liang H., Baxter S.L., McKeown A., Yang G., Wu X., Yan F. (2018). Identifying Medical Diagnoses and Treatable Diseases by Image-Based Deep Learning. Cell.

[8] Khan I.A., Sajeeb A., Fattah S.A. An Automatic Ocular Disease Detection Scheme from Enhanced Fundus Images Based on Ensembling Deep CNN Networks. Proceedings of the 11th International Conference on Electrical and Computer Engineering (ICECE).

[9] Zhang W., Zhao X., Chen Y., Zhong J., Yi Z. (2021). DeepUWF: An Automated Ultra-Wide-Field Fundus Screening System via Deep Learning. IEEE J. Biomed. Health Inform..

[10] Wijesinghe I., Gamage C., Chitraranjan C. Transfer Learning with Ensemble Feature Extraction and Low-Rank Matrix Factorization for Severity Stage Classification of Diabetic Retinopathy. Proceedings of the 2019 IEEE 31st International Conference on Tools with Artificial Intelligence (ICTAI).

[11] Cazañas-Gordón A., Parra-Mora E., Cruz L.A.D.S. (2021). Ensemble Learning Approach to Retinal Thickness Assessment in Optical Coherence Tomography. IEEE Access.

[12] Hendira W.F., Phan Q.T., Adzaka F., Jeong C. (2023). Combining transformer and CNN for object detection in UAV imagery. ICT Express.

[13] Shen S., Wang X., Mao F., Sun L., Gu M. (2022). Movements Classification Through sEMG with Convolutional Vision Transformer and Stacking Ensemble Learning. Sensors.

[14] AlDahoul N., Karim H.A., Tan M.J.T., Momo M.A., Fermin J.L. (2021). Encoding Retina Image to Words using Ensemble of Vision Transformers for Diabetic Retinopathy Grading. F1000Research.

[15] Gupta A., Gautam N., Vishwakarma D.K. Ensemble Learning using Vision Transformer and Convolutional Networks for Person Re-ID. Proceedings of the 2022 6th International Conference on Computing Methodologies and Communication (ICCMC).

[16] Ullah W., Hussain T., Ullah F.U.M., Lee M.U., Baik S.W. (2023). TransCNN: Hybrid CNN and transformer mechanism for surveillance anomaly detection. Engineering Applications of Artificial Intelligence. Eng. Appl. Artif. Intell..

[17] Ullah W., Hussain T., Baik S.W. (2023). Vision transformer attention with multi-reservoir echo state network for anomaly recognition. Inf. Process. Manag..

[18] Yao C., Feng L., Kong Y., Xiao L., Chen T. (2023). Transformers and CNNs fusion network for salient object detection. Neurocomputing.

[19] Yang L., Yang Y., Yang J., Zhao N., Wu L., Wang L., Wang T. (2022). FusionNet: A Convolution–Transformer Fusion Network for Hyperspectral Image Classification. Remote Sens..

[20] Nanni L., Lumini A., Loreggia A., Formaggio A., Cuza D. (2022). An Empirical Study on Ensemble of Segmentation Approaches. Signals.

[21] Zhang Y., Liu H., Hu Q. (2021). TransFuse: Fusing Transformers and CNNs for Medical Image Segmentation. arXiv.

[22] Wang T., Lan J., Han Z., Hu Z., Huang Y., Deng Y., Zhang H., Wang J., Chen M., Jiang H. (2022). O-Net (2022): A Novel Framework With Deep Fusion of CNN and Transformer for Simultaneous Segmentation and Classification. Front. Neurosci..

[23] Szegedy C., Vanhoucke V., Ioffe S., Shlens J., Wojna Z. Rethinking the Inception Architecture for Computer Vision. Proceedings of the 2016 IEEE Conference on Computer Vision and Pattern Recognition (CVPR).

[24] Wen L., Li X., Gao L. (2020). A Transfer Convolutional Neural Network for Fault Diagnosis Based on ResNet-50. Neural Comput. Appl..

[25] Dosovitskiy A., Beyer L., Kolesnikov A., Weissenborn D., Zhai X., Unterthiner T., Dehghani M., Minderer M., Heigold G., Gelly S. (2020). An Image is Worth 16x16 Words: Transformers for Image Recognition at Scale. arXiv.

[26] He K., Zhang X., Ren S., Sun J. Deep residual learning for image recognition. Proceedings of the IEEE Conference on Computer Vision and Pattern Recognition.

[27] Trockman A., Kolter J.Z. (2022). Patches Are All You Need?. arXiv.

[28] Vaswani A., Shazeer N., Parmar N., Uszkoreit J., Jones L., Gomez A.N., Kaiser Ł., Polosukhin I. (2017). Attention is all you need. Advances in Neural Information Processing Systems 30 (NIPS 2017).

[29] Kermany D., Zhang K., Goldbaum M. (2018). Large Dataset of Labeled Optical Coherence Tomography (OCT) and Chest XRay Images. Mendeley Data.

[30] Drummond C., Holte R.C. (2003). C4.5, Class Imbalance, and Cost Sensitivity: Why Under-Sampling Beats Over-Sampling. Workshop on Learning from Imbalanced Datasets II.

[31] Gholami P., Roy P., Parthasarathy M.K., Lakshminarayanan V. (2020). OCTID: Optical Coherence Tomography Image Database. Data.

[32] Li F., Chen H., Liu Z., Zhang X., Wu Z. (2020). Fully automated detection of retinal disorders by image-based deep learning. Graefes Arch. Clin. Exp. Ophthalmol..

[33] Fang L., Jin Y., Huang L., Guo S., Zhao G., Chen X. (2020). Iterative Fusion Convolutional Neural Networks for Classification of Optical Coherence Tomography Images. Sensors.

